# *Leishmania infantum* Defective in Lipophosphoglycan Biosynthesis Interferes With Activation of Human Neutrophils

**DOI:** 10.3389/fcimb.2022.788196

**Published:** 2022-04-06

**Authors:** Graziele Quintela-Carvalho, Astrid Madeleine Calero Goicochea, Vanessa Mançur-Santos, Sayonara de Melo Viana, Yasmin da Silva Luz, Beatriz Rocha Simões Dias, Milena Lázaro-Souza, Martha Suarez, Camila Indiani de Oliveira, Elvira M. Saraiva, Cláudia I. Brodskyn, Patrícia T. Veras, Juliana P.B. de Menezes, Bruno B. Andrade, Jonilson Berlink Lima, Albert Descoteaux, Valéria M. Borges

**Affiliations:** ^1^ Instituto Gonçalo Moniz, Fundação Oswaldo Cruz (FIOCRUZ), Salvador, Brazil; ^2^ Faculdade de Medicina, Universidade Federal da Bahia (UFBA), Salvador, Brazil; ^3^ Instituto Federal de Educação, Ciência e Tecnologia Baiano (IFBaiano), Alagoinhas, Brazil; ^4^ Departamento de Imunologia, Laboratório de Imunobiologia das Leishmanioses, Instituto de Microbiologia Paulo de Góes, Universidade Federal do Rio de Janeiro, Rio de Janeiro, Brazil; ^5^ Escola Bahiana de Medicina e Saúde Pública, Salvador, Brazil; ^6^ Multinational Organization Network Sponsoring Translational and Epidemiological Research (MONSTER) Initiative, Salvador, Brazil; ^7^ Curso de Medicina, Faculdade de Tecnologia e Ciências, Salvador, Brazil; ^8^ Universidade Salvador (UNIFACS), Laureate Universities, Salvador, Brazil; ^9^ Núcleo de Agentes Infecciosos e Vetores (NAIVE), Universidade Federal do Oeste da Bahia (UFOB), Barreiras, Brazil; ^10^ Institut National de la Recherche Scientifique (INRS)–Centre Armand-Frappier Santé Biotechnologie, Laval, QC, Canada

**Keywords:** lipophosphoglycan, *Leishmania infantum*, neutrophils, ROS, infection

## Abstract

Visceral leishmaniasis (VL) is often associated with hematologic manifestations that may interfere with neutrophil response. Lipophosphoglycan (LPG) is a major molecule on the surface of *Leishmania* promastigotes, which has been associated with several aspects of the parasite–vector–host interplay. Here, we investigated how LPG from *Leishmania* (*L*.) *infantum*, the principal etiological agent of VL in the New World, influences the initial establishment of infection during interaction with human neutrophils in an experimental setting *in vitro*. Human neutrophils obtained from peripheral blood samples were infected with either the wild-type *L. infantum* (WT) strain or LPG-deficient mutant (*∆lpg1*). In this setting, *∆lpg1* parasites displayed reduced viability compared to WT *L. infantum*; such finding was reverted in the complemented *∆lpg1*+*LPG1* parasites at 3- and 6-h post-infection. Confocal microscopy experiments indicated that this decreased survival was related to enhanced lysosomal fusion. In fact, LPG-deficient *L. infantum* parasites more frequently died inside neutrophil acidic compartments, a phenomenon that was reverted when host cells were treated with Wortmannin. We also observed an increase in the secretion of the neutrophil collagenase matrix metalloproteinase-8 (MMP-8) by cells infected with *∆lpg1 L. infantum* compared to those that were infected with WT parasites. Furthermore, collagen I matrix degradation was found to be significantly increased in *∆lpg1* parasite-infected cells but not in WT-infected controls. Flow cytometry analysis revealed a substantial boost in production of reactive oxygen species (ROS) during infection with either WT or *∆lpg1 L. infantum*. In addition, killing of *∆lpg1* parasites was shown to be more dependent on the ROS production than that of WT *L. infantum*. Notably, inhibition of the oxidative stress with Apocynin potentially fueled *∆lpg1 L. infantum* fitness as it increased the intracellular parasite viability. Thus, our observations demonstrate that LPG may be a critical molecule fostering parasite survival in human neutrophils through a mechanism that involves cellular activation and generation of free radicals.

## Introduction

Visceral leishmaniasis (VL), also known as Kala-azar, is a reemerging neglected tropical disease spread throughout Asia, Europe, the Middle East, Africa, and the Americas, with an average of 400,000 cases per year (OPAS and WHO, 2016). In the Old World, VL is caused by parasites of the *Leishmania donovani* complex, while in Brazil, *Leishmania infantum* is its main etiological agent (Franssen et al., 2020). Clinically, the disease caused by this parasite is systemic and associated with chronic immunopathology, which causes approximately 59,000 deaths annually worldwide (OPAS and WHO, 2016; Franssen et al., 2020). The lack of a timely adequate treatment leads to disease progression with involvement of organs such as bone marrow, liver, and spleen. Lethality is usually associated with hepatosplenomegaly, persistent fever, weight loss, hematological manifestations, and immunosuppression, which is hallmarked by substantial neutropenia and high susceptibility to death caused by bacterial co-infections (Chappuis et al., 2007). Understanding the interplay between *Leishmania* parasites and host immune cells, and especially neutrophils, may help to develop innovative strategies to optimize patient management and reduce the disease burden.

Neutrophils play a key role in establishing *Leishmania* infection, although macrophages are the major host cells (Peters et al., 2008; Chaves et al., 2020). Neutrophils are the first phagocytic cells to migrate and arrive in the parasite inoculation site during transmission from the invertebrate vectors (Laskay et al., 2003; Laskay et al., 2008; Ribeiro-Gomes and Sacks, 2012). Upon arrival at the infection site, these innate immune cells promote a boost in inflammation through production and/or secretion of a variety of enzymes that result in tissue remodeling, such as the metalloproteinase-8 (MMP-8) (Dieffenbach et al., 2021) , and of reactive oxygen species (ROS) that promote not only parasite killing but also immunopathology (Díaz-Gandarilla et al., 2013; Ferraz et al., 2015). When parasites are phagocytized, neutrophils rapidly release lysosomal enzymes in the intracellular compartment containing *Leishmania*, which results in parasite killing. It is critical for the *Leishmania* parasite to evade neutrophil microbicide responses (Hurrell et al., 2016; Passelli et al., 2021).

Lipophosphoglycan (LPG) is localized at the surface of *Leishmania* promastigotes (Forestier et al., 2015) and has been described as a virulence factor (Forestier et al., 2014; Podinovskaia and Descoteaux, 2015) that protects the parasite from host-mediated damage. LPG also dampens the cellular activation to favor silent entry of *Leishmania* and benefits its persistence inside infected cells (Becker et al., 2003; Spath et al., 2003; Lodge et al., 2006). During neutrophil infection with *Leishmania*, the presence of LPG has been reported to inhibit the pro-oxidative response (Laufs et al., 2002; Salei et al., 2017) and the fusion of phagocytic vacuoles with lysosomes (Gueirard et al., 2008), favoring the success of infection. LPG from *L. donovani* has also been described to favor parasite viability in the presence of neutrophilic microbicidal mechanisms, such as induction of neutrophil extracellular traps (NETs) (Gabriel et al., 2010). Thus, LPG is thought to be a key component of *Leishmania* promastigotes that sustains parasite viability by evading well-established antimicrobial mechanisms inside neutrophils. Whether this molecule operates similarly in other species of *Leishmania* is still an area of great interest and not fully understood. The present study aimed at filling this gap regarding *L. infantum*, which is mostly responsible for the high burden of VL in South America. To this end, we used an LPG-deficient lpg1-/- mutant, which has been previously developed by our group (Lázaro-Souza et al., 2018). The findings reported here point to an interesting role of LPG that promotes parasite survival through an intricate mechanism that involves neutrophil activation, extracellular matrix degranulation, and oxidative stress.

## Methods

### Ethics Statement

Written informed consent was obtained from all the study participants following the Declaration of Helsinki. The protocol was approved by the Institutional Review Board of the Federal University of Sergipe, Brazil (license number: 04587312.2.0000.0058).

### Obtaining Human Neutrophils

Human blood was obtained from healthy volunteers at the Hemocenter of the State of Bahia (HEMOBA). The isolation of neutrophils was done as previously described (Quintela-Carvalho et al., 2017). The collected blood was added in Polymorphprep medium to obtain the polymorphonuclear cloud, performed according to the manufacturer’s instructions (Axis-ShieldPoc AS, Oslo, Norway). Then, the blood was centrifuged for 30 min at 1,300 RPM at 25°C. After centrifugation, two bands were detectable: the first consisted of mononuclear cells and the second consisted of polymorphonuclear cells. Thus, neutrophils were collected and washed three times with saline at 4°C for 10 min at 1,200 RPM. This method allowed a purified population with about 94% neutrophils. Neutrophils were plated at a concentration of 5 × 10^5^ per well, in 96-well plates, with RPMI 1640 medium supplemented with Nutritional-SP 1% and 1% L-glutamine (2 mM), 100 U/ml penicillin, and 100 μg/ml streptomycin.

### Parasite Cultures

Wild-type (WT) *L. infantum* BA262 promastigotes and the isogenic LPG-deficient mutant of *L. infantum* (Lázaro-Souza et al., 2018) were cultured in HOMEM medium supplemented with 10% inactivated fetal bovine serum (FBS), 100 U/ml penicillin, 100 μg/ml of streptomycin, and 2 mM of L-glutamine, at neutral pH and in culture bottles (3 ml or 5 ml of medium) kept inside a BOD at 25°C. The *∆lpg1* parasites were grown in HOMEM medium supplemented with Hygromycin B 50 μg/ml and G418 70 g/ml to prevent LPG from being synthesized (Lázaro-Souza et al., 2018). For infections, WT and *∆lpg1 L. infantum* promastigotes were used in a stationary growth phase.

### Infection of Human Neutrophils

Human neutrophils were plated at a concentration of 5 × 10^5^ per well and were incubated with WT or *∆lpg1 L. infantum* stationary promastigotes, with a 1:10 infection rate (neutrophil:promastigote). The cultures were incubated for 3 h to evaluate the infection.

### Parasite Burden and Viability of Promastigotes *L. infantum* in Infected Human Neutrophils

The phagocytosis of WT, ∆*lpg1*, or ∆*lpg1+LPG1 L. infantum* promastigotes was analyzed through the frequency (%) of neutrophils with internalized parasites, examined by microscopy. The total number of parasites counted inside infected neutrophils (metric: parasites/200 neutrophils), was also examined by optical microscopy at 3- and 6-h post-infection. Moreover, the percentage of infected neutrophils containing CFSE-labeled parasites was assessed using flow cytometry (Fortessa, BD). The viability of promastigotes was assessed by counting viable parasites using an adaptation of the Schneider method (Ribeiro-Gomes et al., 2004; Quintela-Carvalho et al., 2017). Specifically, neutrophils were incubated with WT, ∆*lpg1*, or ∆*lpg1+LPG1 L. infantum* promastigotes for a time course of 3- and 6-h post-infection maintained in a CO_2_ incubator at 37°C with 5% CO_2_ in the presence of RPMI medium with 10% BFS. After 3-h and 6-h post-infection, infected neutrophils were centrifuged at 1,300 RPM for 10 min at 4°C and the supernatant containing non-internalized promastigotes was discarded and replaced with 200 μl of HOMEM medium. Then, the infected neutrophils were incubated in the BOD at 22°C, and after 24 h, viable promastigotes that form freely in the medium were counted in Neubauer chambers. During this time, *Leishmania* parasites proliferate/multiply extracellularly in HOMEM medium, thus explaining why the total number of parasites at the 24-h time point is higher than that observed in the initial inoculum.

For membrane fusion inhibition assay, promastigote viability in infected neutrophils was assessed in 30 min pretreatment with Wortmannin (500 nM - SIGMA).

### Transmission Electron Microscopy

After 3 h of infection with WT and *∆lpg1 L. infantum* promastigotes, neutrophils were washed, collected, and fixed in 0.1 M cacodylate buffer, pH 7.2, with 1% glutaraldehyde (Sigma-Aldrich), 4% formaldehyde, and 5 mM CaCl_2_. Subsequently, they were post-fixed in 1% osmium tetroxide and 0.08% potassium ferricyanide. The dehydration was done in increasing series of acetone concentration and replaced by Polybed resin (Polysciences Inc., USA). After being cut into ultrafine sections, the cells were stained in uranyl acetate and citrate for observation under a Zeiss EM 109 transmission electron microscope and recording of representative images.

### Confocal Immunofluorescence Microscopy

For the immunofluorescence assays, WT and *∆lpg1 L. infantum* promastigotes were labeled with probe 5(6)-Succinimidyl carboxyfluorescein ester (Cell Trace CFSE Cell Proliferation Kit) at a concentration of 2 μM, for 10 min and washed 3 times in PBS 1X buffer to remove excess CFSE. Human neutrophils were infected with WT or *Δlpg1* parasites labeled with CFSE and incubated with Lysotracker red for acid compartments localization (Molecular Probes). After 3 h of infection, nuclear staining was done with 4’,6-diamidino-2-phenylindole (DAPI) and the samples were observed through a fluorescence confocal microscope (Leica Microsystems). The number of lysotracker positive vacuoles was quantified in 10 distinct fields and the spots represent individual cells containing colocalization of lysotracker and *Leishmania* positive vacuoles.

### Quantification of MMP-8

To analyze the release of neutrophilic enzyme MMP-8, supernatants from controls and cultures of infected neutrophils with WT or *Δlpg1 L. infantum* promastigotes were collected and immediately tested for the presence of enzyme, according to the manufacturer’s instructions (R&D systems, Minneapolis, USA).

### Matrix Degradation Assay

Collagen type I matrix (Collagen I, Rat Tail - Gibco) was prepared at a 2 mg/ml concentration with 10% PBS phosphate buffer 10X, 0.26% NaOH 1 N and 10% fluorescent gelatin (Gelatin from pigskin, Oregon green 488 conjugate – Invitrogen). Neutrophils (5 × 10^4^) were then added to the matrix and cultured for 3 h at 37°C and 5% CO_2_, fixed with PFA 4% for 15 min, and visualized with a Leica DMi8 inverted fluorescence microscope. Quantification of matrix degradation was assessed by measuring the pixels of gelatin-FITC degradation area using FIJI software. Areas corresponding to 30 cells were quantified for each condition in 3 separate experiments.

### Detection and Inhibition of Oxidative Response

To evaluate oxidative response inhibition, human neutrophils were previously incubated or not with 20 μM of Apocynin (Sigma), a potent antioxidant (REF), for 1 h, followed by 30 min of infection with WT or ∆lpg1 *L. infantum* promastigotes. After this period, these cultures were treated for an additional 30-min interval with 10 μM of the dihydroethidium probe (DHE) (Invitrogen/Molecular Probes, Grand Island, NY, USA). Using this assay, upon entering cells, a DHE probe is oxidized by superoxide anions emitting red fluorescence and detected by flow cytometry (Fortessa, BD).

### Statistical Analysis

Statistical analyses were performed using the GraphPad Prism 8.0 (GraphPad Software, San Diego, CA, USA). Infection experiments were performed in quintuplicate, and data on central tendency and dispersion are presented as medians and interquartile ranges, respectively. Comparisons were made using the nonparametric Mann–Whitney *U* test or the Kruskal–Wallis test (for more than two samples) with Dunn’s multiple comparisons post-test. Frequencies (%) of categorical variables were compared using the Pearson’s chi-square test. Differences were considered statistically significant when *p* < 0.05.

## Results

### *LPG1*-Null Mutants Exhibit Limited Survival in Neutrophils

**Figure 1:**
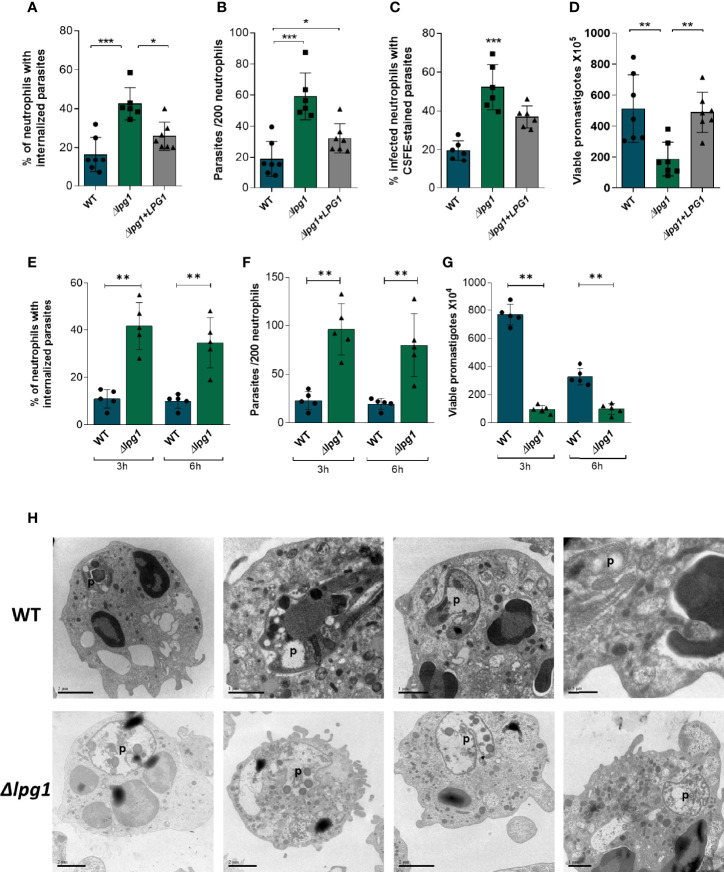
Viability and ultrastructure of *Lpg1-null* mutants in neutrophils. Human neutrophils were infected with *L. infantum* WT, *∆lpg1*, or *∆lpg1+LPG1* for 3 h Light microscopy was used to assess the percentage of neutrophils that contained internalized parasites **(A)** and number of parasites per 200 neutrophils **(B)**. Flow cytometry was used to examine the percentage of neutrophils containing CSFE-labeled parasites **(C)**. In similar experiments, human neutrophils were infected for 3 h, when the culture medium was replaced by HOMEM medium. Count of released viable promastigotes in supernatant was performed after 24 h **(D)**. Each point in the graphs represents a donor; bars represent median values and whiskers infer interquartile ranges. Asterisks indicate statistically significant differences assessed by the non-parametric Kruskal–Wallis test with Dunn’s multiple comparisons *ad hoc* test (*p < 0.05, **p < 0.01, ***p < 0.001). Human neutrophils were infected with *L. infantum* WT or *∆lpg1* (1:10) for 3 and 6 h Light microscopy was used to assess the percentage of neutrophils that contained internalized parasites **(E)** and the number of parasites per 200 neutrophils **(F)**. In similar experiments, human neutrophils were infected for 3 and 6 h when the culture medium was replaced by HOMEM medium. The number of released viable promastigotes in the supernatant was obtained after 24 h **(G)**. Each dot in the graphs represents a donor; bars represent median values, and whiskers infer interquartile ranges. Asterisks indicate statistically significant differences evaluated through the non-parametric Mann–Whitney *U* test (**p < 0.01). Human neutrophils were infected for 3 h with *L. infantum* WT or *∆lpg1*, processed and analyzed by transmission electron microscopy (TEM); representative images are shown **(H)**.

To determine the role of LPG during infection, human neutrophils were infected for 3 h with WT, *∆lpg1*, and *∆lpg1* + *LPG1 L. infantum* promastigotes. We performed assays counting frequency (%) of infected cells (**Figure 1A**) and the number of parasites per cell by microscopy (**Figure 1B**). Moreover, we used flow cytometry to determine the % of infected cells with CFSE-labeled parasites (**Supplementary Figure 1** and **Figure 1C**). The survival assays revealed that *∆lpg1* parasites presented impaired viability inside neutrophils compared to that detected with wild-type *Leishmania* (**Figure 1D**). We also performed an infection comparing an early (3 h) and a later (6 h) time point where most of the parasites were already internalized. The percentage of infected cells (**Figure 1E**) and number of parasites per 200 cells (**Figure 1F**) were similar at both time points, indicating that *∆lpg1* parasites are more frequently internalized by neutrophils. In addition, parasite survival was analyzed and we observed that regardless of the time of infection (3 h or 6 h), the *∆lpg1* parasites presented impaired viability inside neutrophils compared to that detected with WT *Leishmania* (**Figure 1G**). Furthermore, morphology of neutrophils infected with WT or *∆lpg1 L. infantum* promastigotes was analyzed by transmission electron microscopy (TEM). We noted that the cellular structure and morphology of WT *L. infantum* in the parasitophorous vacuole were well preserved. In contrast, *∆lpg1* parasites exhibited disturbed morphology with degradation of intracellular structures, which may be indicative of parasite killing (**Figure 1H**). These results show that the WT parasites and the *∆lpg1* + *LPG1* complemented parasites behave similarly. For this reason, we performed the next experiments with the WT and *∆lpg1* mutant parasites only. These results show that *∆lpg1 L. infantum* parasites infect more neutrophils than WT parasites, but they do not survive as much as the WT once they are internalized. The findings suggest that LPG may be important for parasite persistence inside infected cells.

### LPG Hampers Lysosomal Fusion and Formation of Acidic Compartments Containing *L. infantum*

**Figure 2:**
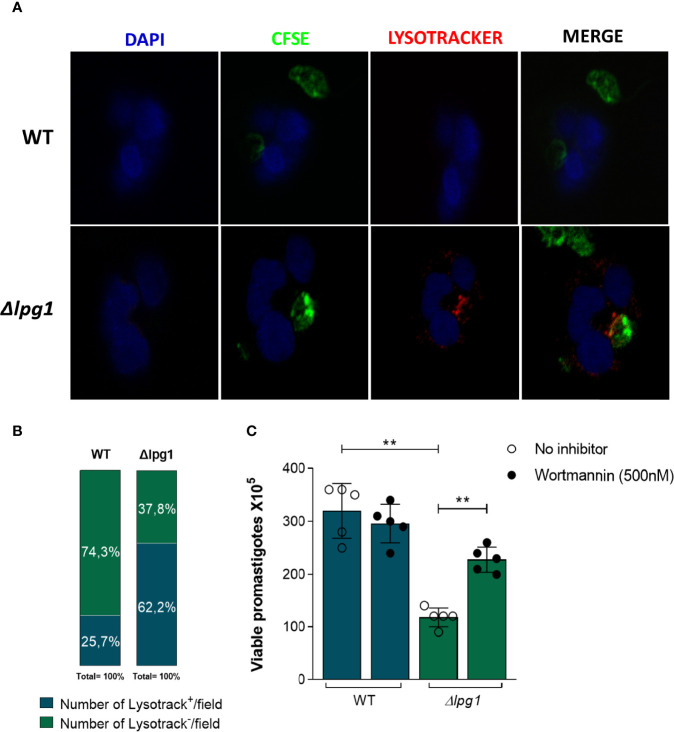
Impact of neutrophils’ *acidic* compartments on survival of *Lpg1*-*null* mutant parasites. **(A)** Human neutrophils were infected with *L. infantum* WT or *∆lpg1* stained with carboxyfluorescein succinimidyl Ester (CFSE) in green and incubated with Lysotracker (lysosomal district marker) in red and with DAPI (nuclear mark) in blue. After 3 h, acidic parasitophorous vacuoles in neutrophils were imaged for colocalization using a fluorescence microscopy, and representative data are shown **(A)**. Frequency of neutrophils staining positive for Lysotracker per microscopy field was compared between the groups of cells infected with WT or *∆lpg1* parasites. Data were compared using the Pearson chi-square test (*p* < 0.05) **(B)**. Human neutrophils, treated or not with Wortmannin (500 nM), were infected with *L. infantum* WT or *∆lpg1* for 3 h, when RPMI was replaced by HOMEM medium. Count of released viable promastigotes in supernatant was performed after 24 h **(C)**. Each point in the graphs represents a donor; bars represent median values and whiskers infer interquartile ranges. Asterisks indicate statistically significant differences evaluated through the non-parametric Mann–Whitney *U* test (**p < 0.01).

The results reported above indicated that viability of *∆lpg1* parasites was diminished compared to that observed in WT controls. This finding led us to hypothesize that a potential mechanism underlying such phenomenon may implicate lysosomal activity. To test this idea, human neutrophils were infected with WT or *∆lpg1 L. infantum* promastigotes labeled with CFSE (green fluorescence) followed by Lysotracker staining (red fluorescence). After 3 h of infection, we observed that *∆lpg1 L. infantum* mutant co-localized with lysotracker whereas WT controls did not (**Figure 2A**). Reinforcing the information shown in confocal microscopy, the quantitative results indicated that the frequency of lysotracker-positive vacuoles/field was substantially higher in cultures of neutrophils infected with *∆lpg1* parasites compared to those of cells infected with WT controls (**Figure 2B**). To confirm that trafficking of *∆lpg1* parasites to lysosomes was involved with the impairment of their viability, we repeated the viability assay in the presence of Wortmannin, an inhibitor of parasitophorous vacuole acidification (Tavares et al., 2016). Inhibition of neutrophil vacuole acidification led to an increased viability of *∆lpg1 L. infantum* parasites, which is consistent with the notion that LPG may protect *L. infantum* from degradation in acidic compartments (**Figure 2C**).

### *L. infantum* Genetically Lacking *LPG1* Activates Human Neutrophils to Secrete MMP-8

**Figure 3:**
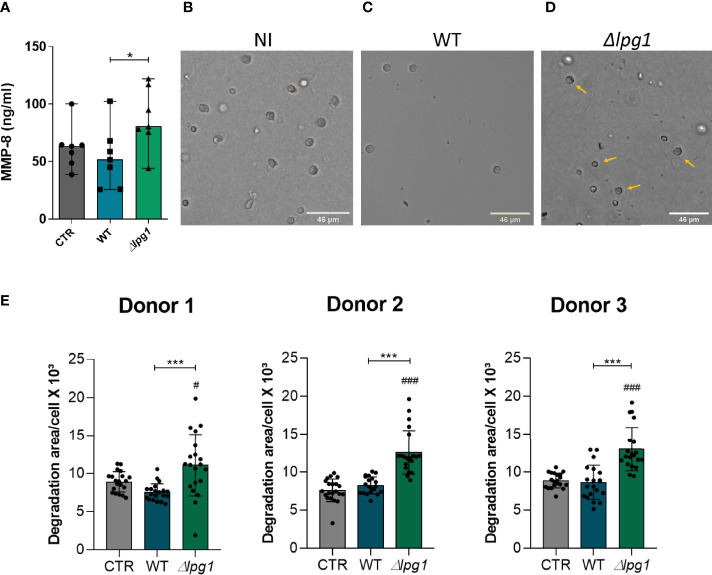
Infection with *L. infantum ∆lpg1* triggers secretion of MMP-8 and extracellular matrix type-I collagen degradation. Human neutrophils were infected with *L. infantum* WT or *∆lpg1* for 3 h Concentrations of MMP-8 in cell supernatants were quantified by ELISA and compared between the groups **(A)**. Representative images of collagen I matrix degradation by human neutrophils in the following conditions: non-infected **(B)**, infected with WT **(C)**, or infected with *∆lpg1 L. infantum* **(D)**. Pictures were obtained using an inverted microscope. Quantification of collagen I matrix degradation was performed using the FIJI program, from 20 to 25 cells per group, which are represented by the points on the graph; data from three distinct experiments using cells from different donors are shown **(E)**. In **(A)** Asterisk indicate significant differences examined using the nonparametric Mann–Whitney U test (*p < 0.05,). In **(E)** Asterisks indicate statistically significant differences assessed by the non-parametric Kruskal–Wallis test with Dunn’s multiple comparisons ad hoc test (***p < 0.001).Differences between experimental group and control are indicated with hashtags (^#^p < 0.05) and (^###^p < 0.001).

We next tested whether the absence of LPG could be driving neutrophil activation. MMP-8 is an important product of neutrophil activation and has a role in acute inflammation through collagen degradation (Dieffenbach et al., 2021). We found higher concentrations of MMP-8 in culture supernatants of neutrophils infected with *∆lpg1 L. infantum* compared to uninfected cells or those infected with WT *controls* (**Figure 3A**). To delineate the potential consequences of this augmented secretion of MMP-8 by neutrophils infected with *∆lpg1 parasites*, we performed a type-I collagen matrix degradation assay in our experimental model. The microscopy images confirmed the reduction of the collagen matrix in cultures of neutrophils infected with *∆lpg1 L. infantum* (**Figures 3B–D**). **Figure 3E** summarizes the experiments to show that neutrophils infected with the ∆*lpg1 L. infantum* mutant displayed, on average, a higher matrix degradation area compared to control or WT-infected neutrophils (**Figure 3E**). Although there were statistically significant differences in MMP-8 and collagen degradation measures between the experimental groups, the magnitude of such differences may be seen as low at a first glance. Increases of 30% in degranulation of enzymes that degrade extracellular matrix are likely to be relevant biologically; however, our experimental system does not allow us to investigate such matter *in vivo*. Regardless of such limitations, these results argue that LPG may indeed interfere with the production of neutrophil-associated matrix remodeling.

### Death of *L. infantum ∆lpg1* Promastigotes Is Dependent on ROS Production

**Figure 4:**
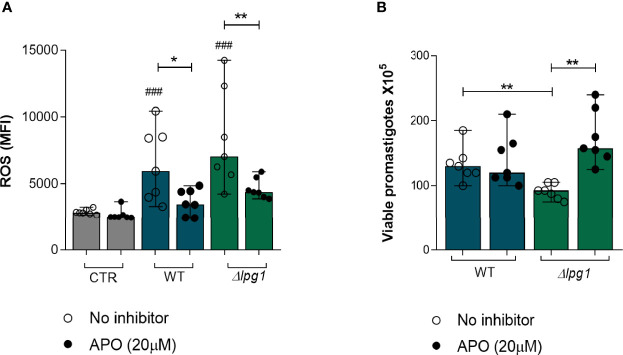
Death of *L. infantum ∆lpg1* promastigotes is dependent on ROS production. Human neutrophils were treated or not with Apocynin (APO) (20 µM) for 1 h, followed by 30 min of infection with *L. infantum* WT or *∆lpg1*. After this period, neutrophils were incubated with the dihydroethidium probe (DHE) and ROS production was analyzed using flow cytometry **(A)**. Human neutrophils, treated or not with Apocynin (20 µM), were infected with *L. infantum* WT or *∆lpg1* for 3 h, when culture medium was replaced by HOMEM medium. Count of released viable promastigotes in supernatant was performed after 24 h **(B)**. Each point in the graphs represents a donor; bars represent median values and whiskers infer interquartile ranges. Asterisks indicate significant differences examined using the nonparametric Mann–Whitney *U* test (*p < 0.05, **p < 0.01). Differences between indicated experimental group and control are indicated with ### (*p* < 0.001).

Although the results so far have demonstrated a role for LPG in protecting *L. infantum* promastigotes from the microbicidal activity of neutrophils and in dampening neutrophil activation, the specific mechanism leading to the killing of the *∆lpg1* mutant parasites in neutrophils had not been clarified. ROS are well-known microbicidal mediators against bacteria and parasites (Díaz-Gandarilla et al., 2013; Forrellad et al., 2019). We therefore evaluated the potential role of ROS in the killing of *∆lpg1 L. infantum* promastigotes. First, we measured ROS production by neutrophils infected with WT or *∆lpg1 L. infantum* promastigotes in the presence or not of a potent ROS inhibitor (Apocynin). The results demonstrated that both WT and *∆lpg1* parasites induced ROS production to a similar extent (**Figure 4A**); such effect was reverted by the treatment with Apocynin, as expected. Since our results reported above indicated that LPG protects promastigotes from the microbicidal activity of neutrophils, we next investigated whether this protection occurs through affecting sensitivity to ROS. We performed a parasite viability assay in the absence or the presence of the ROS inhibitor Apocynin. As shown in **Figure 4B**, the presence of Apocynin in cultures of neutrophils infected with the WT promastigotes had no effect on the viability of the parasites. Strikingly, inhibition of ROS production led to substantial increase in the viability of the *∆lpg1 L. infantum* parasites. These findings agree with our hypothesis that *∆lpg1 L. infantum* are more sensitive to oxidative stress responses from neutrophils.

## Discussion

Lipophosphoglycan, the most abundant glycoconjugate on the surface of *Leishmania* promastigotes, has been shown to play a central role in the ability of these parasites to establish infection in the host phagocytes (Ribeiro-Gomes & Sacks, 2012). Results from previous studies with LPG-deficient *∆lpg1* mutants in *L. donovani* or other species from the Old World cannot always be validated in species from the New World (Privé and Descoteaux, 2000). In our study, we investigated how LPG from *L. infantum*, the principal etiological agent of VL in Brazil, influences the initial establishment of infection during interaction with human neutrophils.

Using a well-established *in vitro* model with infection of human neutrophils, we observed that the LPG-deficient *L. infantum* parasites are more frequently phagocytized when compared to LPG-expressing WT or *∆lpg1* + *LPG1* parasites. However, the results from the viability assay indicate that once internalized, the LPG-defective parasites do not survive at 3 h post infection to the same extent as that observed with WT or *∆lpg1* + *LPG1* parasites. This observation suggests that this molecule is important for the ability of the parasite to survive and establish infection in human neutrophils. In studies performed in the past (Ribeiro-Gomes and Sacks, 2012; Tavares et al., 2014; Quintela-Carvalho et al., 2017), we detailed analysis of the dynamicity of human neutrophil infection, activation, and death *in vitro*. In our hands, in the context of the experiments with *Leishmania* infection *in vitro*, neutrophils do not survive for much longer after isolation from peripheral blood (after 8 h post isolation since these cells undergo apoptosis). In addition, neutrophils are leukocytes with extremely rapid responses, and several important mediators are immediately released from endovesicles without the need of *de novo* production. Therefore, it is not possible to evaluate infectivity after 8 h post-infection without a high degree of bias due to death of neutrophils. For this reason, our experiments were carried out at 3 h and 6 h post infection.

Since the abovementioned results were similar to experiments of internalization and viability with WT and *∆lpg1* + *LPG1* parasites, we performed the next assays only comparing the WT with the LPG-deficient *L. infantum* parasites. When combined, our experiments indicate that LPG from *L. infantum* may be a factor dampening neutrophil activation. Thus, LPG-lacking parasites may more robustly activate neutrophils, which would explain increased phagocytosis, infection, and intracellular killing. Our findings prompted us to hypothesize that such effect of LPG on human neutrophils may be a critical mechanism to foster parasite persistence and establishment of infection in susceptible hosts.

A previous observation that an antibody against LPG prevented *L. major* promastigote binding to macrophages (Handman and Goding, 1985) led to the conclusion that LPG is required for promastigote internalization. However, with the availability of genetically defined LPG-defective mutants generated in both *L. major* and *L. donovani*, it became clear that LPG is completely dispensable for promastigote internalization by macrophages (Holm et al., 2003; Spath et al., 2003). In fact, as reported by both Spath et al. (2003) and Holm et al. (2003), uptake of LPG-defective promastigotes by macrophages is superior to that of WT and add-back parasites. Further investigation revealed that LPG reduces the phagocytic capacity of macrophages by excluding the membrane fusion regulator Synaptotagmin (Syt) V from the nascent phagocytic cup (Vinet et al., 2011). Syt V regulates phagocytosis by controlling focal exocytosis of endocytic organelles (Vinet et al., 2008). Our results showing increased internalization of LPG-defective *L. infantum* by neutrophils are thus consistent with the previous findings described above.

To survive intracellularly, vacuolar pathogens have developed mechanisms to evade the action of microbicidal molecules acting inside vesicles of phagocytic cells. In macrophages, bacteria such as *Mycobacterium tuberculosis* or protozoa such as *Toxoplasma* and *Leishmania* can survive by preventing the formation of microbicidal phagolysosomes (Robert-Gangneux and Dardé, 2012; Forrellad et al., 2013; EDR and JE, 2016). Here, we investigated whether LPG interferes in a similar mechanism to protect *L. infantum* promastigotes within parasitophorous vacuoles in human neutrophils. Our observation that *∆lpg1 L. infantum* parasites, but not WT, are found within acidified parasitophorous vacuoles is consistent with previously reported findings demonstrating that LPG prevents phagolysosome biogenesis and acidification (Desjardins and Descoteaux, 1997; Dermine et al., 2000; Gueirard et al., 2008; Vinet et al., 2009; da Silva Vieira et al., 2019; Matte et al., 2021). To confirm the role of acidified compartments in the killing of *∆lpg1 L. infantum*, we performed a viability assay in the presence of Wortmannin, an inhibitor of vesicle fusion and phagolysosome formation (Tavares et al., 2016). Wortmannin treatment during neutrophil infection increased the viability of *∆lpg1 L. infantum* to a similar degree of that quantified in cells infected with WT *L. infantum*. Notably, these observations are in agreement with previous investigations using promastigotes from *Leishmania major* and *L. donovani* species (Dias et al., 2018; Verma et al., 2018). In those experiments, promastigotes survived in human neutrophils by preventing the early fusion of specific and tertiary granules with the vacuole containing the parasite. This result reinforces the idea that LPG plays a protective role to favor *Leishmania* persistence inside neutrophils through a mechanism that involves phagosome fusion. Wortmannin is a potent PI3K inhibitor, and its effects on diminishing phagosome acidification are very well described (Krysko et al., 2006; Cheekatla et al., 2012), but this drug may have pleiotropic effects and thus further investigations are required to address whether the effects described in our study are reproducible with other and more selective inhibitors of phagosomal acidification.

Neutrophil microbicidal mechanisms to control infections include the release of granule contents in the phagosome or extracellularly (Carlsen et al., 2015; Hurrell et al., 2016). We took two approaches to investigate this phenomenon in the context of our experimental model. We first examined whether infection was related with release of MMP-8, which represents an important neutrophil-associated collagenase. The experiments indicated that the concentration of the neutrophilic enzyme MMP-8 was increased in supernatants of neutrophils infected with *∆lpg1 L. infantum* when compared to ones infected with the WT.

MMPs are a family of proteolytic enzymes related to tissue remodeling and inflammation (Sivak and Fini, 2002). Some MMPs may be associated with damage to the extracellular matrix (Nusblat et al., 2011). To test whether increased MMP-8 release would result in potential tissue remodeling, we performed a matrix degradation assay. As expected, neutrophils infected with *∆lpg1 L. infantum* degraded a more extended area of matrix compared with uninfected cells or those infected with WT parasites. This augmented matrix degradation is a hallmark of tissue remodeling observed during infection of host tissues and thus our results argue that LPG may restrict tissue damage. We hypothesize that LPG contributes to a more silent infection and less disturbance of host homeostasis, which will minimize the inflammatory response against the parasite.

The second line of investigation of the microbicidal mechanisms was to define whether LPG also interferes with oxidative effector functions. Neutrophil activation status is directly associated with its ability to produce reactive oxidative species. Several pathogens such as *Leishmania* can induce or potentiate the oxidative response in neutrophils (Laufs et al., 2002; Carlsen et al., 2015). We measured ROS production by neutrophils using flow cytometry. The results indicated that infection with either WT or *∆lpg1 L. infantum* promoted a similar increase in ROS production by neutrophils. Thus, LPG does not seem to directly affect pro-oxidation promoted by neutrophil activation during infection. Previous work from our group has demonstrated that the reduction of *∆lpg1 L. infantum* survival during infection of murine macrophages is related to higher levels of NF-κB-dependent iNOS induction, which drives nitric oxide generation (Lázaro-Souza et al., 2018). In macrophages, the LPG from *Leishmania* has been associated with reduction in superoxide levels as it impairs the assembly of the NADPH complex (Lodge et al., 2006). Neutrophils infected with *L. major* are also known to decrease oxidative stress in the presence of apoptotic cells, promoting the persistence of the parasite in these cells (Mollinedo et al., 2010; Salei et al., 2017). Our findings suggest that the oxidative response could contribute to the control of the parasite load in neutrophils infected with *∆lpg1 L. infantum* promastigotes, mainly because these parasites do not present LPG on its surface as a protective barrier.

*Leishmania*’s LPG is thought to inhibit the maturation of vacuoles in which they are contained by blocking recruitment of the v-ATPase and acidification (Vinet et al., 2009; Matte et al., 2021) and assembly of the NADPH oxidase (Lodge et al., 2006). The blockage of phagosome acidification inhibits action of ROS. As the results so far indicated that LPG was acting through a similar mechanism in our model, we speculated that *∆lpg1 L. infantum* is not capable of preventing phagosome maturation, causing an increased ROS production in neutrophils that could be reversed in the use of NADPH oxidase inhibitors. To test this hypothesis, we used Apocynin during the infection of neutrophils with *∆lpg1 L. infantum*. The results observed in **Figure 4B** show that APO treatment had no effect on parasite viability in cultures infected with WT but it did improve viability of the *∆lpg1* mutant. Thus, diminished ROS seem to be contributing more to survival of the LPG-defective strain in this experimental setting than that of WT. The results cannot delineate why WT parasites were less sensitive to ROS inhibition with APO, but they do show that there was a robust effect on *∆lpg1* parasites. Ideally, one could perform the assays in cells KO to NADPH oxidase, which could clarify the role of ROS in the system without the use of inhibitors and would also allow the identification of the role of host-derived ROS. It is possible that the presence of LPG in WT parasites has promoted escape mechanisms that involve resistance to ROS inside infected cells, minimizing the odds of detecting an effect of the Apocynin treatment. Altogether, these data support the role of LPG in the protection of *L. infantum* from ROS microbicidal effect.

The combined findings exposed here uncover novel nuances about the role of *L. infantum* LPG as a relevant virulence factor that interferes with the capacity of the neutrophils to promote successful parasite killing. By exerting a negative effect on phagosome fusion, neutrophil activation, and oxidative metabolism, LPG may support survival of parasites inside host cells, which is a critical path towards persistence of infection and parasitism. Further studies are necessary to investigate the pathways and other mechanisms involved in the role played by the activation of neutrophils and other cell types.

## Data Availability Statement

The raw data supporting the conclusions of this article will be made available by the authors, without undue reservation.

## Ethics Statement

The protocol was approved by the Institutional Review Board of the Federal University of Sergipe, Brazil (license number: 04587312.2.0000.0058). The patients/participants provided their written informed consent to participate in this study.

## Author Contributions

Conceptualization: GQ-C, AG, VM-S, BA, JL, AD, and VB. Methodology: GQ-C, AG, VM-S, SM, YS, BD, ML-S, MS, CO, ES, CB, PV, JM, BA, JL, AD, and VB. Data analysis: GQ-C, AG, VM-S, SM, YS, BD, ML-S, MS, CO, ES, CB, PV, JM, BA, JL, AD, and VB. Writing: GQ-C, AG, VM-S, BA, JL, AD, and VB. Funding acquisition: AD and VB. All authors contributed to the article and approved the submitted version.

## Funding

This work was supported by the Brazilian National Research Council (CNPq, 431857/2018-0) and Programa Inova – Geração de Conhecimentos (FIOTEC, VPPCB-007-FIO-18-2-101) to VB and the Canadian Institutes of Health Research (CIHR) (grant PJT-156416 to AD). CO, ES, CB, PV, BA, and VMB are senior investigators funded by CNPq. AD is the holder of the Canada Research Chair on the Biology of intracellular parasitism. AG received a fellowship from the FAPESB and VM-S received a fellowship from the CNPq. This study was financed in part by the Coordenação de Aperfeiçoamento de Pessoal de Nível Superior - Brasil (CAPES) - Finance Code 001. The funders had no role in study design, data collection or analysis, the decision to publish, or preparation of the manuscript.

## Conflict of Interest

The authors declare that the research was conducted in the absence of any commercial or financial relationships that could be construed as a potential conflict of interest.

## Publisher’s Note

All claims expressed in this article are solely those of the authors and do not necessarily represent those of their affiliated organizations, or those of the publisher, the editors and the reviewers. Any product that may be evaluated in this article, or claim that may be made by its manufacturer, is not guaranteed or endorsed by the publisher.

## Supplementary Material

Supplementary Figure 1 Gating strategy of human neutrophils infected with CFSE-stained *L. infantum.* Parasites pre-stained with Carboxyfluorescein Succinimidyl Ester (CFSE) were used to infect Neutrophils for 3 h. **(A)** Cells were first selected in a sideward scatter channel (SSC) vs. forward scatter channel (FSC) plot, then singlets were identified by FSC-H vs. FSC-A plot for exclusion of debris and live cells were selected based on negative Fixable Viability Dye staining. Subsequently, the gating of infected cells was set on the positive CFSE population. **(B)** Individual plots showing representative populations of neutrophils uninfected, or infected with *L. infantum* WT, ∆lpg1 or ∆lpg1+LPG1, respectively.
