# Supplementary figures and images for: Leishmania infantum Defective in Lipophosphoglycan Biosynthesis Interferes With Activation of Human Neutrophils
Source: Front Cell Infect Microbiol. 2022 Apr 6;12:788196. doi: 10.3389/fcimb.2022.788196 (PMC9019130; doi:10.3389/fcimb.2022.788196)

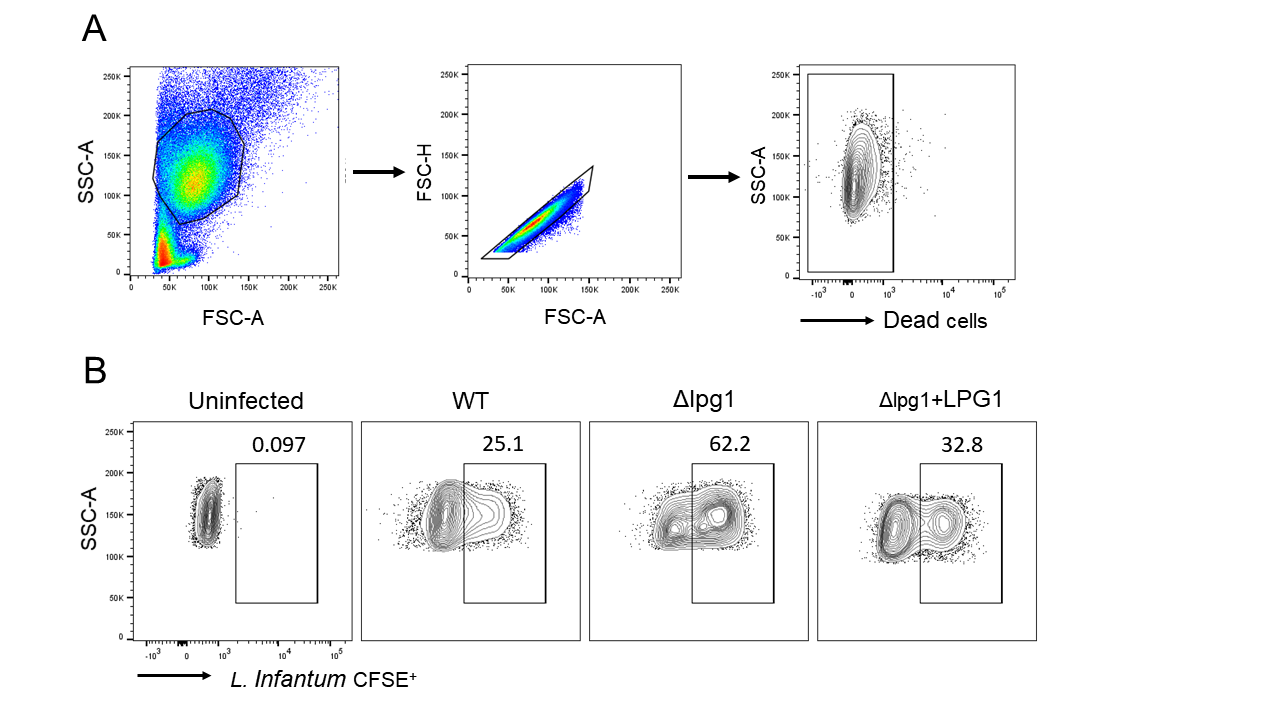

Supplement: Supplementary Figure 1 — Gating strategy of human neutrophils infected with CFSE-stained L. infantum. Parasites pre-stained with Carboxyfluorescein Succinimidyl Ester (CFSE) were used to infect Neutrophils for 3 h. (A) Cells were first selected in a sideward scatter channel (SSC) vs. forward scatter channel (FSC) plot, then singlets were identified by FSC-H vs. FSC-A plot for exclusion of debris and live cells were selected based on negative Fixable Viability Dye staining. Subsequently, the gating of infected cells was set on the positive CFSE population. (B) Individual plots showing representative populations of neutrophils uninfected, or infected with L. infantum WT, ∆lpg1 or ∆lpg1+LPG1, respectively. [file Image_1.tif]
